# The environmental effects of the “twin” green and digital transition in European regions

**DOI:** 10.1007/s10640-022-00741-7

**Published:** 2022-12-09

**Authors:** Stefano Bianchini, Giacomo Damioli, Claudia Ghisetti

**Affiliations:** 1 BETA, Université de Strasbourg, Strasbourg, France; 2 Joint Research Centre, European Commission, Ispra, Italy; 3 Faculty of Business Studies and Economics, University of Bremen, Bremen, Germany; 4 CAPP – Centre for the Analysis of Public Policies, University of Modena and Reggio Emilia, Modena, Italy; 5 Department of Sociology and Social Research, University of Milan-Bicocca, Milan, Italy

**Keywords:** Technological innovation, GHG emissions, Twin transition, Digital ecosystem, O30, Q53, Q55, R11

## Abstract

This study explores the nexus between digital and green transformations—the so-called “twin” transition—in European regions in an effort to identify the impact of digital and environmental technologies on the greenhouse gas (GHG) emissions originating from industrial production. We conduct an empirical analysis based on an original dataset that combines information on environmental and digital patent applications with information on GHG emissions from highly polluting plants for the period 2007–2016 at the metropolitan region level in the European Union and the UK. Results show that the local development of environmental technologies reduces GHG emissions, while the local development of digital technologies increases them, albeit in the latter case different technologies seem to have different impacts on the environment, with big data and computing infrastructures being the most detrimental. We also find differential impacts across regions depending on local endowment levels of the respective technologies: the beneficial effect of environmental technologies is stronger in regions with large digital technology endowments and, conversely, the detrimental effect of digital technologies is weaker in regions with large green technology endowments. Policy actions promoting the “twin” transition should take this evidence into account, in light of the potential downside of the digital transformation when not combined with the green transformation.

## Introduction

The recent emergence of (advanced) digital technologies has generated a new wave of optimism that a wide range of social, economic, and environmental goals might at last be realized, including that of sustainable, inclusive growth. Yet, at the same time, concerns have been expressed about the potentially adverse effects of the pervasive diffusion of these technologies, including rising unemployment (Brynjolfsson and Mitchell 2017), growing inequality and discrimination (O'Neil 2016), the emergence of dysfunctional democracies (Zuboff 2019), and, even, uncontrolled human-enhancement (Bostrom 2017). One of the more controversial issues to emerge with some force is the *environmental impact* they might have on a world that is becoming increasingly digital (Vinuesa et al. 2020; Coeckelbergh 2021a; del Río Castro et al. 2021); this is unfortunate, as we stand on the threshold of a green transition promoted by a series of innovative policy actions.

Indeed, in the scientific community and the policy arena, as well as in the press, a number of legitimate questions have begun to circulate: Are the green and digital transitions mutually compatible? Or is one transition likely to cancel out the other? These are the overarching questions we address in this study. While the term “transition” is broad, spanning the economic, social, cultural, and political spheres, here we focus our attention on its technological dimension. More specifically, this study examines the effects that green technologies and a combination of advanced digital technologies—additive manufacturing, artificial intelligence, big data, computing infrastructures, internet of things (IoT), and robotics—have on the greenhouse gas (GHG) emissions of industrial production in European regions.

The large-scale economic activity of the last two centuries has changed the Earth’s climate, its biodiversity, water and nitrogen cycles, and ocean chemistry. According to some climate scientists, such is the severity of this damage that we are beginning to exceed the planet’s “safe operating limits” and we have begun to cross some of the “planetary boundaries” beyond which we venture at our peril (Rockstrom et al. 2009; Steffen et al. 2015). One of the main concerns here is human-induced climate change, the result of massive GHG emissions into the atmosphere originating from burning coal, oil, and gas for “making things” (cement, steel, and plastics), “plugging in” (electricity), “growing things” (plants and animals), “getting around” (planes, trucks, and cargo ships), and “keeping warm and cool” (heating, cooling and refrigeration) (Gates 2021, p. 55).

These GHG emissions warm the planet and usher in a new climate. Indeed, we are currently living in the “Anthropocene”, a term coined to define this geological epoch, in which humanity has had a dramatic impact on the Earth’s physical and biological systems, turning humans into a geological force (Crutzen 2006; Lewis and Maslin 2015), and in which a combination of “adaptation” and “mitigation” strategies has become a pressing need. While adapting to life in a changing climate will be a necessity, mitigating GHG emissions is now a priority to limit global warming and one that is made blatantly clear in both the 2030 Agenda for Sustainable Development (UN 2015)—“*Roadmap for redefining sustainable development as a people and planet agenda: A prosperous and fair world within the planetary boundaries*” (TWI^2050^,^2019^, p. 7)—and in the European Union’s (EU) 2020 Industrial Strategy, which sets out explicit directions for a globally competitive, climate-neutral, and digitalised economy—i.e., the green and digital transitions that were quickly baptized the *“twin”* transition (EC 2020a, 2020b; Bauer et al. 2021).^1^

Technology plays a central role in most strategies designed to cope with climate change. Green technology know-how offers several solutions for the transition to a low-carbon economy, ranging from carbon (air and point) capture, e-fuels, and advanced biofuels to zero-carbon cement, plastic, and steel technologies. A comprehensive literature shows that these technologies can effectively reduce the environmental impact of production.

There are also high expectations—but, as yet, no systematic evidence – that digital technologies can help tackle environmental problems (see, e.g., Rolnick et al. 2019). Artificial intelligence (AI) and big data, for instance, can positively contribute to the study of climate change by detecting new patterns in environmental data (EC 2020c; Vinuesa et al. 2020); by nudging consumers to behave in more climate-friendly ways and increasing awareness of their environmental footprint (Coeckelbergh 2021a); by promoting smart, low-carbon cities by interconnecting electric vehicles, smart appliances, and smart grids for energy management and routing (del Río Castro et al. 2021); and by guiding policy actions aimed at reducing emissions, preserving the environment and predicting the occurrence of hazardous events such as heat waves and storms (Onyango and Ondiek 2021). However, we should not be overly simplistic about the redemptive impact of ‘going digital’; it is evident that there is a negative side to technology too. Afterall, it is heavily dependent on energy, infrastructure, and materials (Jones 2018; Strubell et al. 2019) and, as a result, its net effects are controversial.

The complexity of the interplay between green and digital technologies, and their environmental effects, calls for a new research agenda, and it is the assessment of this need that constitutes the main objective of the present study. To the best of our knowledge, our contribution is original in this regard, being the first study to attempt such an analysis. The second element of originality characterizing our study is the empirical analysis we report of the impact of both digital and green technologies on the GHG emissions of industrial production at a highly granular level, namely that of the European NUTS 3 metropolitan areas. In this way, we are better able to accommodate the spatial dimension of environmental performance (Gibbs and O’Neill 2017; Dong et al. 2018), knowledge creation (Audretsch and Feldman 2004), and technological production (Alcacer and Chung 2007). The third innovative step taken by our study is the use we make of an original dataset that combines patent applications and emission data. More specifically, we draw on green patent applications registered with the European Patent Office (EPO) in the OECD REGPAT database; digital EPO patent applications in the EPO-PATSTAT database; and emissions data from the European Pollutant Release and Transfer Register (E-PRTR), covering roughly 45% of total GHG emissions from production activities. We then aggregate patent and emission data at the level of 1051 metropolitan regions (henceforth, metro-regions), ensuring a granular level of aggregation for the econometric analysis, and resulting in a sample of 10,510 observations for the period 2007–2016.

Based on this sample, we estimated a set of econometric models to assess the environmental returns of digital and green (local) technology development, while accounting for the endogeneity between green technology and emissions, by using instrumental variables. The results of the analysis indicate that the local development of environmental technologies significantly reduces emissions at highly polluting plants. In contrast, the local development of digital technologies has a mixed impact: on the one hand, digital technologies directly increase emissions, due, in all likelihood, to their high-energy requirements and to waste disposal, yet, on the other, the interaction of environmental and digital technologies positively contributes to emission abatement; however, the latter effect only partly offsets their direct adverse effects. Additionally, we find that different digital technologies appear to have different environmental impacts, with big data and computing infrastructures being the most detrimental.

The rest of the paper is organized as follows. Section 2 outlines the conceptual framework of our research. Section 3 describes the empirical approach, including the construction of the dataset and the econometric strategy. Section 4 presents and discusses the main results of the analysis. Section 5 concludes and offers a number of policy implications.

## Conceptual Framework

This study contributes to ongoing discussions about the role of the so-called “twin” transition in facilitating climate change mitigation (see, among others, Vinuesa et al. 2020; Coeckelbergh 2021b; del Rìo Castro et al. 2021). Here, we specifically focus on the technology dimension of the twin transition by assessing the relationship between the development of green and digital technologies and the release of GHG emissions from industrial activities.

Only a small number of studies have undertaken an empirical examination of the “twin” transition and even fewer have adopted a granular technological or regional lens. Cicerone et al. (2022), for example, explore the enabling role of AI for regional specialisation in green technologies. Montresor and Vezzani (2022) draw on data from an Italian survey to show that firms’ investments in AI support their ability to adopt environmental innovations. However, no study, to date, has investigated the environmental effects of the technologies underpinning the “twin” transition.

In this section, we review the literature in order to develop empirically testable hypotheses about this, as yet, under-investigated relationship.

### Technology, Innovation and Environmental Performance

Technology is widely considered one of the core determinants of the environmental impact of human activities. Since the early 1970s, scholars have systematically assessed anthropogenic impacts on the environment, resulting in the development of the well-known IPAT identity framework (Commoner et al. 1971; Ehrlich and Holdren 1971) with technology very much at the heart of the matter.

The IPAT identity models global environmental impacts (I) as the multiplicative effect of population (P), affluence (A) and technology (T), and has been further extended to capture the “stochastic impacts by regression on population, affluence, and technology” (i.e., the STIRPAT decomposition model), whereby the drivers of degradation are decomposed to evaluate their individual impact on the environment. Overall, the existing literature points to a set of potential determinants of environmental degradation, that constitute, at the very minimum, income per capita, population, technology, energy consumption, and the structure of the economy. The literature on the IPAT or STRIPAT models is vast and encompasses analyses conducted at different levels (global, macro, meso and micro), focused on different countries and leading mostly to confirmatory results (Dietz and Rosa 1994; York et al. 2003). Complementary to the IPAT approach, studies examining the environmental Kuznets curve (EKC) (Andreoni and Levinson 2001) find theoretical and empirical support for the presence of an inverted U-shaped relationship between environmental degradation (mostly conceived in terms of various indicators, including air pollution but also water and soil pollution or waste generation) and economic development (mostly measured in terms of per capita income). This branch of the literature explains the decoupling of degradation *vis-à-vis* development that occurs after a certain turning point, recognizing, again, the central role played by advances in technology after a particular level of economic development has been achieved. Further extensions show an N-, rather than a U-, shaped, relationship for certain sectors and pollutants, but they still serve to corroborate the crucial role played by technology (Marin and Mazzanti 2013).

Most empirical studies confirm the importance of innovation and technology for emission abatement, supporting the hypothesis that (green) innovations contribute to carbon emission reduction, at different levels of analysis: the *macro-level*, including the EU-27 (Töbelmann and Wendler 2020), G7 (Wang et al. 2020; Khan et al 2020), OECD (Ganda 2019; Alvarez-Herranz et al. 2017; Hashmi and Alam 2019), ASEAN (Salman et al. 2019), BRICS (Khattak et al. 2020), and multiple countries (Du et al. 2019; Chen and Lee 2020); the *meso/sectoral-level*, both in multiple (Costantini et al. 2017) and single countries, including the UK (Cole et al. 2005), the US (Carrion-Flores and Innes 2010), Spain (Tarancòn and del Rio 2007) and Italy (Ghisetti and Quatraro 2017); and the *micro-level*, for instance among US (Shadbegian and Grey 2003) and Japanese firms (Cole et al. 2013; Lee and Min 2015).^2^

Previous research points to the need to focus on the regional characteristics of emission data, as there appear to be regional specificities among the factors affecting local carbon emissions. Most regional studies have been conducted in the Chinese provinces or regions and confirm the role played by inventive activities in curbing emissions (Luan et al. 2019; Liang et al. 2019; Wang and Zhu 2020; Zhang et al. 2020, 2017). Zheng et al. (2020), for instance, in their study of different industries in 30 Chinese regions between 2007 and 2016, show carbon emissions increase differently across regions, an outcome they attribute to industrial structure, economic growth, population, and urbanization. In Europe, Costantini et al. (2013) undertake a sector-regional analysis of Italian NUTS 2 regions and find that technology improves the environmental performance of a place and that regional knowledge spillovers are critical to this improvement.

Not only does the existing literature suggest that regional characteristics (including innovation) directly affect regional environmental performance, but it also shows that the regional dimension is crucial in shaping the (green or dirty) dimension of technology change which, in turn, has an indirect effect on emissions. Regional environmental policies are reported to stimulate the development of green technologies (Ghisetti and Quatraro 2013). Regional environmental expenditure—that is, investments—and environmental management are, likewise, found to support firm-level innovative activities (D’Agostino and Moreno 2019). Previous regional specialization (or non-specialization) in green technologies is also likely to affect current and future specialization in these technologies, in a process that is strongly path-dependent (Montresor and Quatraro 2020; Santolaha and Boschma 2021).

Although previous research suggests that regional technological capacity (especially in green technologies) can contribute to emission abatement, the fact that the overall net effect will be mediated by choices taken at the micro level (i.e., by the firms themselves) cannot be ignored. Indeed, it is the firms that ultimately choose whether or not to resort to available (greener) technological knowledge. Likewise, firms may, or may not, have acquired sufficient absorptive capacity to benefit from innovation developed elsewhere (Marrucci et al. 2021) and have, or not have, the internal organization to be able to make the effective transition to cleaner production choices, e.g., through dedicated management schemes (Seman et al. 2019). However, it is unclear as to whether or not these relationships hold at the regional level, i.e., to what extent environmental performance is improved by the availability of local green knowledge. Ghisetti and Quatraro (2017), for instance, suggest that regions/sectors characterized by higher inventive activity in environmental technologies are also those that record better environmental performance, even when controlling for inter-sectoral interdependence.

Thus, in line with the above theoretical arguments and empirical evidence, we can expect a positive impact of environmental technologies on GHG emissions or, formally:

#### H1

The (local) development of environmental technologies contributes to reducing (local) GHG emissions originating from production activities.

### The Environmental Footprint of the Digital Transition

There is broad agreement that the ongoing digital transformation is changing the environment; however, the direction of this change is the subject of much debate (Sachs 2020). In common with discussions on many emerging technologies, opinion is polarised: its detractors claim “*digitalisation will destroy the planet*”; its proponents counter “*digitalisation is the solution for environmental sustainability*”. Just where the truth lies is unclear as there has been little research into the environmental consequences of the digital transformation. Moreover, it should be stressed that digital technologies cannot be treated as a uniform entity, but rather as a set of different, often complementary and interconnected, bodies of knowledge—i.e., as a *digital ecosystem*. This means that different digital technologies are likely to exert disparate forces on the environment.

Yet, most digital technologies have certain characteristics in common, not least their high energy footprint. Global digital energy consumption increased by around 9% per annum in the period 2015–2020, and this trend is expected to increase if no immediate action is taken (IEA 2017). The building blocks of the digital ecosystem—big data distribution, storage and use; the computational power required to process such data, for example, by means of machine learning algorithms and neural networks; the connection of devices (IoT); the peripherals and industrial robots being increasingly used in production processes; and, additive manufacturing machine tools—are all high consumers of energy that, in turn, exacerbate GHG emissions (Dusik et al. 2018; Jones 2018; Strubell et al. 2019). In 2018, Joppa and Herweijer (2018) calculated that the share of GHG emissions attributable to digital technologies was set to increase from 2.5% in 2013 to 4% by 2020.^3^

The second characteristic these technologies have in common are certain traits of their life cycle, especially phases of raw material extraction and waste disposal. The mass production of digital equipment, devices and infrastructure *in primis* makes digital technologies heavy intermediate consumers of materials, some of which are rare and whose accessible reserves are limited. To this must be added the use of plastics for manufacturing devices and in their packaging. There are two obvious side effects: first, severe soil pollution during the extraction of materials and GHG emissions during the processing of the latter as input into the production process, though their relevance for the empirical analysis conducted in this study is negligible given that Europe is largely dependent on other countries for raw materials (mainly China, South-East Asia and certain African states), components and assemblies relevant to robotics, additive manufacturing and ICT technologies (European Commission 2020d); and, second, and more importantly for our research, most of the materials are not circular—i.e., they cannot be re-cycled or re-used—leading to a technological dead end and critical waste management issues, again increasing GHG emissions (Shift Project 2019; Kunkel and Matthess 2020).

The negative effects of the digital transition on the environment—let us say the “downside” of digital technologies—allow us to hypothesise that:

#### H2

The (local) development of digital technologies directly contributes to increasing (local) GHG emissions originating from production activities.

But there is also a “bright side” to the effects of digital technologies on the environment; although, they are in the main indirect. In most current policy discourse, at least in Europe, the focus is on the synergies between the digital and green transitions, on the understanding that digital technologies can be used to tackle societal challenges, including those of an environmental nature. A few recent studies characterise digital technologies—in particular, deep learning—as an ‘emerging method of invention’ that can have spillover effects on other innovations and technology breakthroughs across the whole economy (Cockburn et al. 2019; Bianchini et al. 2022), including environmental innovation and technologies. For example, AI-related technologies have been found to help regions that already possess a green specialisation to “remain green” (Cicerone et al. 2022). Moreover, digital technologies can boost firm-level productivity through the automation of production processes and, in turn, enhanced productivity should result in a more efficient use of resources and encourage other forms of investments, such as in green technologies (Antonioli et al. 2018).

In the light of the above discussion, our last research question seeks to understand whether places that are better endowed with environmental technologies benefit not only directly from this endowment (as suggested by H1) but also indirectly. More specifically, we test for the presence of an interaction between environmental and digital technologies, to evaluate whether their joint presence can alleviate the downside of digital technologies and strengthen the benefits associated with environmental technologies or, more formally:

#### H3

The reduction (increase) in GHG emissions from production activities in places with a more advanced development of environmental (digital) technologies is augmented (reduced) by the joint presence of a more advanced development of digital (environmental) technologies.

In the following section, we describe our empirical approach. Given the multidimensional nature of the digital ecosystem, our analysis also seeks to open up the “black box” of digital technologies and unbundle them into their main building blocks. The empirical analysis we describe focuses first on an aggregate category of digital technologies and, then, on its subcomponents, in an effort at understanding *what* digital technology is associated with the bright and down sides of environmental performance.

## Empirical Analysis

Our empirical analysis tests the three hypotheses presented above by applying a regionalised version of the IPAT framework,^4^ where I (*impact*) is measured by the level of regional GHG emissions and is a function of P (*population*), A (*affluence*, approximated by value added), T (*technology*), and a set of additional contingent factors that serve as controls. To shed light on the environmental impacts of the “twin” transition, T (technology) is differentiated into digital and green regional technology capabilities and approximated, as we discuss below, by patent applications in the digital and green domains. Our focus is specifically on technology–environment patterns at the fine-grained level of the metropolitan regions, which—unlike administrative geographical units—allows for a spatially coherent representation of economic activity.^5^

Our choice of geographic level for this analysis responds, in the first place, to the need to consider environmental performance at the sub-national level, given the heterogeneity of energy endowments and development plans across regions (as discussed, for example, in Dong et al. 2018). Earlier green economy–innovation transition research tended to neglect both the spatial aspect and the regional dimension of this transition (Truffer and Conen 2012). More recently, however, a research agenda for regional studies of the green economy has been proposed that is centred more fully on examining the socio-spatial embedding of the conditions needed to support technology development in certain places and the sustainability transition (Truffer and Conen 2012; Truffer et al. 2015), in recognition of the fact that regions (and places, in general) can play a crucial role in shaping this transition (Gibbs and O’Neill 2017).

Second, the geographical level of analysis selected is consistent with the idea that the spatial context is crucial for facilitating innovation capacity and reducing the barriers to innovation. There is a long tradition in so-called “new economic geography” for considering innovative output and technology adoption to be lower in regions characterised by a paucity of economic knowledge, the rationale being that new knowledge—even when highly codified in the form of patents—has an important tacit component. As such, the externalities that this knowledge has across firms and industries are bounded in space and, generally, decay quickly as they move across this geographic space. Indeed, although the costs of transmitting information have fallen substantially with the emergence of ICTs, the marginal costs of transmitting new technological knowledge remain lower in the presence of frequent social interactions, observations, and communication between users and producers (Audretsch and Feldman 2004). Proximity increases the ability of companies to exchange ideas about incipient knowledge, thereby reducing the uncertainty of dealing with new and emerging technologies (Alcacer and Chung 2007).

### Data and Variables

#### GHG Emissions

We build an original dataset of regional emissions of greenhouse gases (GHG) by retrieving information on air emissions from the European Pollutant Release and Transfer Register (E-PRTR) dataset (E-PRTR_database_v17—last update October 2019).

E-PRTR data include all compulsory reports of emissions for the period 2007–2017 by EU Member States, covering about 45% of GHG emissions.^6^ Firms are only obliged to report these emissions if they belong to certain sectors, in the main, highly energy-intensive or highly polluting sectors, such as, energy, metal, chemical, mining, paper, and wastewater treatment—see Appendix A of the Regulation concerning the establishment of the E-PRTR, EC No 166/2006 of the EU Parliament and EU Council of January 2006 provides for a detailed list of sectors. All facilities carrying out one or more of the activities specified in Appendix A are obliged to register emissions when the latter exceeds the applicable capacity thresholds specific to the sub-sector and the substance emitted, as detailed in Appendix B. More specifically, Article 7 of the Regulation requires Member States to submit an annual report detailing releases to air, water, and land as well as all transfers of pollutants in wastewater for 91 substances across 65 industrial sub-sectors, and the transfer of waste from these industrial facilities.

The E-PRTR register includes information for different pollutants for more than 33,000 facilities in 33 countries (EU-28, Iceland, Liechtenstein, Norway, Switzerland, and Serbia). The E-PRTR is based on *real* emission data and provides information on emissions that are imputed to industrial production. This means that no emissions attributable to consumption or transport are included in these data, which is an advantage as far as this study is concerned as we can be relatively confident that the effect of the technologies that we seek to examine is limited to its impact on industrial emissions—i.e., those emissions most likely to benefit from the adoption of green and digital technologies.

Data were assigned to each NUTS 3 based on the exact location (postcode) of the plant responsible for that release. Of the 6876 facilities in the sample of companies required to report GHG emissions, 28% reported data for the entire period, 15% for just one year, and the remaining provided mixed coverage over time. Unfortunately, we have no information as to why a company might have exited the E-PRTR, be it for reasons of bankruptcy, other events (e.g., M&A), or the adoption of innovations that led the facility to reduce its emissions below the threshold and so it was no longer obliged to register. A further limitation of the E-PRTR is that it only records emissions from highly energy-intensive and polluting plants, but ignores all other industrial emissions. This means that some metro-regions in our sample may have no GHG emissions simply because of the demographics of their local facilities. However, none of the available alternatives would have overcome this limitation.^7^

**Fig. 1:**
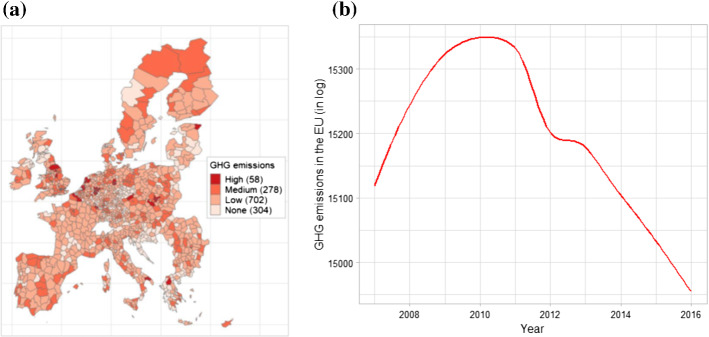
Geographical distribution and time trends of GHG emissions in the European Union, 2007–2016. *Notes:* The categories in panel (**a**) are based on percentiles: x ≤ 75° is low; 75° < x ≤ 95° is medium; x > 95° is high; and the number of metro-regions corresponding to each is included in parentheses. In panel (**b**), emissions have decreased steadily since 2010, which is consistent with general trends throughout Europe (EEA 2021)

Figure 1 (panel a) shows the geographical distribution of GHG emissions for 1051 metro-regions based on E-PRTR data. The map indicates that high and medium levels of GHG emissions are recorded in most European countries, without their presenting any apparent pattern of concentration. If anything, however, we detect a relatively large number of metro-regions responsible for high emissions in Germany, as well as a fair number of metro-regions in Poland, Greece, and Italy. As for changes over time (Fig. 1 panel b), the aggregate EU-27 plus UK trend follows an inverted U-shape pattern, with GHG emissions rising until 2009, remaining virtually stable up to 2011, and steadily falling thereafter.^8^

#### Environmental Technology Capabilities

Patent applications to the European Patent Office (EPO) were identified using the OECD ENV-TECH classification of environmental technologies (OECD 2017). The ENV-TECH classification flags specific International Patent Classes (IPC) and Cooperation Patent Classes (CPC) as “environmental” or “green”. Selected technologies cover patents in the field of environmental management (air, water, waste, and soil pollution management); climate change mitigation (energy generation, transmission or distribution; transport; buildings; production; wastewater treatment); water related adaptation technologies; biodiversity protection; carbon capture and storage.

We retrieved 76,968 green patent applications between 2004 and 2016. In this period, green patents accounted, on average, for between 9% and 11% of all patent applications. Green, as well as digital patents, were geo-localised based on the applicant’s address and assigned, under a full counting scheme, to the corresponding region(s). Full, rather than fractional counting, was employed on the grounds that patents measure technological knowledge and represent regional capabilities. Indeed, when seen from this perspective, it is irrelevant as to whether the knowledge embedded in a patent is shared with other regions. Additionally, we considered applicants from large companies in the EU, rather than individual inventors, in an effort at capturing as closely as possible technology capabilities feeding into regional economic production.^9^

**Fig. 2:**
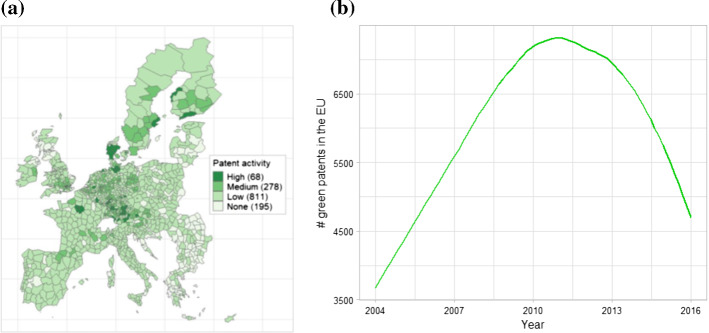
Geographical distribution of environmental patents (per 100,000 inhabitants) and time trends in the European Union, 2004–2016. *Notes:* Environmental patent applications to the European Patent Office between 2004 and 2016 with at least one applicant located in the European Union. The categories in panel (**a**) are based on percentiles: x ≤ 75° is low; 75° < x ≤ 95° is medium; x > 95° is high; and the number of metro-regions corresponding to each is included in parentheses

Figure 2 (panel a) shows the geographical distribution of green patents across metro-regions normalised by population. Compared to the spatial distribution of GHG emissions, green patents are much more concentrated geographically, particularly in Central and Northern metro-regions, while their presence is marginal in Eastern and Southern regions. As for changes over time (Fig. 2 panel b), we observe a rapid increase at the beginning of the period, peaking in 2010/11, followed by a decline in recent years. Indeed, this decline has brought the annual number of patents down to the levels recorded in the early 2000s. Inventive activity appears to have undergone something of a deceleration across all major environment-related technology domains (data not shown here). This slowdown would appear to have two complementary explanations: on the one hand, the increased volatility of energy prices in recent years and, on the other, the great uncertainty regarding the direction and ambition of environmental and climate policies at both national and global levels (for an in-depth discussion, see OECD 2017).

#### Digital Technology Capabilities

The ongoing digital transformation is typically understood as the economic and societal effects of a homogeneous set of technologies, with a particular emphasis on AI (Di Vaio et al. 2020; Goralski and Tan 2020; Truby 2020; Vinuesa et al. 2020). While this somewhat simplistic view has its attractions, it is more appropriate to take a holistic view and consider a much broader spectrum of interconnected technologies, each differing in scope, life cycle, and degree of adoption and diffusion—that is, a digital ecosystem. This ecosystem is more complex, much stronger, and more functional than its individual components given that the latter interoperate with and complement one another (OECD 2019).

Digital technologies are often classified in macro categories that include AI, big data, IoT, 3D printing, and others. Yet, defining the boundaries of these categories is far from easy, and the literature has yet to agree upon a common approach. Studies that seek to capture digital-related innovations have, to date, relied upon hierarchical patent classification systems (e.g., IPC and CPC) (Ardito et al. 2018; Fujii and Managi 2018), keyword inclusion/exclusion criteria for text fields (Webb 2018; Van Roy et al. 2020), or a combination of the two (WIPO 2019; Martinelli et al. 2021). Keywords and technology classes are often heterogeneous—exhaustive lists in some cases, extremely limited in others—although there are some indispensable classes that are present in virtually all studies: “Robotics”, “Neural Networks”, “Big Data” to name a few.

In this study, we define the boundaries of the digital ecosystem and its macro-categories using a comprehensive list of search terms largely inspired and enriched by OECD taxonomy (2019, p. 18), recent contributions on AI mapping (Van Roy et al. 2020; Bianchini et al. 2022) and a set of components of the Industry 4.0 paradigm (Martinelli et al. 2021). Thus, the digital ecosystem in our study includes the following categories: *Additive Manufacturing*; *Artificial Intelligence*; *Big Data*; *Computing Infrastructures*; *Internet of Things*; and *Robotics.* Appendix A provides a more comprehensive description of these six macro-components.^10^

As Table 1 highlights, our search terms are broad in scope, referring to both generic techniques applicable in various contexts (e.g., neural networks) and more specific applications (e.g., rapid prototyping). We are aware that boundaries are blurred and may change over time as technologies (co)evolve, eventually giving rise to new technologies. Yet, the taxonomy proposed here can be deemed instrumental in capturing the general trends.

**Table 1:** Search terms for the identification of digital patents

Additive manufacturing	Artificial intelligence	Big data	Computing infrastructures	Internet of things	Robotics
3D print*	Artificial intellig*	Apache Hive	Cloud application*	Connected device*	Autonomous car*
3D prototyping	Automated reasoning	Apache Kafka	Cloud architecture*	Connected home*	Autonomous underwater vehicle*
Additive fabrication	Backpropagation	Apache Spark	Cloud broker	Cyber-physical system*	Autonomous vehicle*
Additive layer manufacturing	Computer vision	Apache Cassandra	Cloud client	Human–machine interface*	AUV
Additive manufacturing	Data mining	Big data	Cloud computing	Hyper connectivity	Chatbot*
Binder jetting	Data science	BigTable	Cloud infrastructure*	Industrial internet of things	Cybernetics
Composite filament fabrication	Deep learning	Data center*	Cloud migration	Intelligent factor*	Drone*
Continuous liquid interface production	Expert system*	Data centre*	Cloud optimizer	Internet of everything	Humanoid robot*
Direct digital manufacturing	Face detection	Distributed file system*	Cloud platform*	Internet of things	Manipulator*
Direct ink writing	Feature extraction	Docker	Cloud portfolio	IoT	Mobile manipulator*
Direct metal laser sintering	Generative adversarial network*	Hadoop	Cloud provider	Machine-to-enterprise	Mobile robot*
Directed energy deposition	Gesture recognition	HBase	Cloud server*	Machine-to-human	Robot*
Directed energy deposition	Image classification	Large-scale data*	Cloud service*	Machine-to-machine	Robotic*
Electron-beam freeform fabrication	Image recognition	MapReduce	Cloud sourcing	Pervasive sensing	Self-driving car*
Electron-beam melting	Image segmentation	Massive data*	Cloud storage	Sensor network*	Self-driving vehicle*
Fused deposition modeling	Information retrieval		Cluster computing	Smart device*	UAV
Fused filament fabrication	Intelligent machine*		Cognitive comput*	Smart factor*	UGV
Laminated object manufacturing	Kernel machine		Community cloud*	Smart home*	Uncrewed vehicle*
Layered manufacturing	Knowledge representation		Cyberinfrastructure*	Smart sensor*	Unmanned aerial vehicle*
Material extrusion	Machine intelligence		Data-intensive comput*	Wearable*	Unmanned air vehicle*
Material jetting	Machine learning		Decentralized computing	Wireless body area network*	Unmanned aircraft system*
Powder bed fusion	Machine translation		Dew Computing	Wireless sensor network*	Unmanned ground vehicle*
Rapid prototyping	Meta-learning		Distributed computing		Unmanned spacecraft
Robocasting	Multilayer perceptron*		Dynamic cloud*		Unmanned underwater vehicles*
Selective laser melting	Natural language processing		Edge computing		Unmanned vehicle*
Selective laser sintering	Neural net*		Federated cloud*		
Sheet lamination	Object detection		Fog computing		
Stereolithography	Object identification		Grid computing		
Vat photopolymerization	Object recognition		Hardware accelerator*		
	Pattern recognition		High performance comput*		
	Pose estimation		Hybrid cloud*		
	Reinforcement learning		Infrastructure as a service		
	Semantic search		Inter-cloud comput*		
	Semi-supervised learning		Massively parallel processing		
	Sentiment analysis		Multi-cloud*		
	Speech recognition		Neuromorphic comput*		
	Statistical learning		On-demand computing		
	Supervised learning		Optical comput*		
	Text classification		Parallel computing		
	Transfer learning		Photonic comput*		
	Transformer network*		Platform as a service		
	Unsupervised learning		Private cloud*		
	Voice recognition		Public cloud*		
			Quantum comput*		
			Real-time comput*		
			Software as a service		
			Supercomput*		

As a common practice, * after a keyword implies the search strategy included any possible wording or suffix

**Fig. 3:**
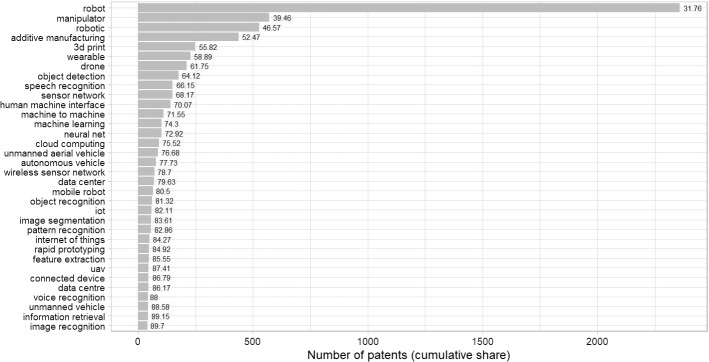
Most recurrent search terms in the digital patent corpus

Patent families were retrieved from the EPO-PATSTAT database. Our query returned a total of 6222 patent applications between 2004 and 2016, a significantly smaller volume than that of green patents (more than tenfold). A large share of these patents is related to robotics, 3D printing and machine learning applications (Fig. 3).

**Fig. 4:**
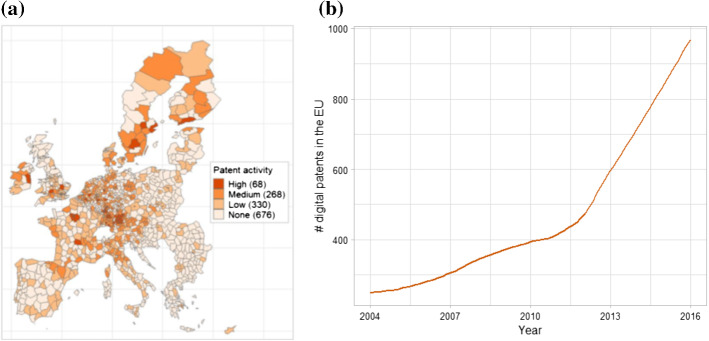
Geographical distribution of digital patents (per 100,000 inhabitants) and time trends in the European Union, 2004–2016. *Notes:* Digital patent applications to the European Patent Office between 2004 and 2016 with at least one applicant located in the European Union. The categories in panel (**a**) are based on percentiles: x ≤ 75° is low; 75° < x ≤ 95° is medium; x > 95° is high; and the number of metro-regions corresponding to each is included in parentheses

Figure 4 (panel a) shows the geographical distribution of digital patents across metro-regions normalised by population. Digital patents present the same strong core-periphery divide observed for green patents, but the distribution is generally much sparser, with a few “digital leaders” in most Western and Northern European countries. Southern Europe (apart from some areas in Northern Italy and Spain) and, especially, Eastern Europe, lag behind with patent activity largely absent in most Eastern metro-regions (676 metro-regions have no patent activity vs. 196 in the case of environmental patents). As for changes over time (Fig. 4 panel b), we observe a distinctive pattern of increasing patenting activity, with a pronounced acceleration in the most recent period.^11^ Taken together, the spatial and growth patterns of the technologies considered confirm that they are in a relatively early stage of their life cycle, compared to the green technologies which present a much stronger degree of maturity.

### Econometric Model

Our model takes the following form:

1 $$\begin{aligned} GHG_{i;t} &= \alpha + \beta_{1} Green \;Tech_{i;t,t - 2} + \beta_{2} Digital\;Tech_{i;t,t - 2} + \beta_{3} \left( {Green\;Tech_{i;t, t - 2} * Digital\;Tech_{i;t, t - 2} } \right) \\ & \quad + \gamma Controls_{i;t} + \delta Pre \;Sample \;Mean\; GHG_{i} + \tau \;Time_{t} + \varepsilon_{i;t} \\ \end{aligned}$$

where *i* indexes the 1051 metropolitan regions and *t* indexes time, the ten years between 2007 and 2016, giving us a working sample of 10,510 observations. The dependent variable *GHG* measures greenhouse gas emissions from production activities measured in kg/year. Our main explanatory variables *Green Tech.* and *Digital Tech.* are EPO environmental and digital patent applications with at least one applicant based in region *i*. We reduce the volatility typical of innovative processes and their outcomes, including patenting activities, by considering a 3-year moving average of patent applications in time *t*, *t −* 1 and *t − *2. Control variables (*Controls*) include the regional population (*Population*) from the Eurostat database; the degree of urbanisation of the metro-regions measured using three dummy variables (*Rural*, *Intermediate* and *Urban*, the last of these serving as the benchmark category in the estimation) based on the Eurostat and DG REGIO taxonomy; the share of value added in the manufacturing sector (*Value Added Manufacturing*) from the Cambridge Econometrics regional dataset; and the amount of units in energy intensive sectors^12^ of the NUTS2 region a metropolitan area belongs to, extracted from Eurostat Structural Business Statistics.^13^

We used Tobit regression to account for the censored nature of our dependent variable. Also, we included a regional fixed-effect of emissions in the pre-sample period (*Pre-sample Mean GHG*), computed as the average of the emission values over the first two years available in E-PRTR data—i.e., 2001 and 2004. This covariate enables us to capture persistent unobserved regional differences in emissions, which may be due, for instance, to regional preferences for sustainable goals, “dirtier” regional economic structures disproportionately more reliant on highly polluting sectors than others, or greater overall path-dependence in emissions accounting for the regional emission trajectories (Blundell et al. 2002; Nesta et al. 2014). Finally, $\beta_{1}$, $\beta_{2}$, $\beta_{3}$, $\gamma$, $\delta$ and $\tau$ denote parameters, or a vector of parameters in the case of $\gamma$ and $\tau$, to be estimated.

In line with H1, H2 and H3, we expect $\beta_{1} < 0$ (i.e., environmental technology capabilities reduce the GHG emissions from production activities), $\beta_{2} > 0$ (i.e., digital technology capabilities increase the GHG emissions from production activities) and $\beta_{3} < 0$ (i.e., beneficial effects of environmental technologies on the GHG emissions from production activities are reinforced by digital technologies or, conversely, the detrimental effects of digital technologies are mitigated by environmental technologies).

All continuous variables are log-transformed. We employ a static analysis, rather than a dynamic panel estimation, because of the nature of our emission data. In fact, when we decompose the variance of GHG emissions, it is evident that most of it (76%) occurs between units, only 7% is inter-temporal and the rest is within-unit and -time variance.

Summary statistics and pairwise correlations are reported in Tables 5 and 6 in Appendix B.

### Instrumental Variable Approach

Previous research hints at the presence of a reverse causality bias in the innovation–emission nexus, as stronger regional competences for environmental technologies can modify emissions, but, at the same time, emissions are important factors driving technology uptake. This means local pollution (or an increase in emission rates) can stimulate technological improvements and inventive activities aimed precisely at developing environmental technologies (Carriòn-Flores and Innes 2010; Lin and Zhu 2019; Ghisetti and Quatraro 2013, 2017; Wang et al. 2020; Lin and Zhu 2019). Additionally, we cannot rule out an omitted variable bias, given that existing policies may positively induce technology change and, also, directly abate emissions (Albrizio et al. 2017). To account for environmental policies, we included as a covariate the OECD Environmental Policy Stringency composite indicator (Botta and Kozluk 2014); however, this variable was found not to be significant (leading to its elimination), but we cannot rule out the possibility that environmental policies continue to be an omitted variable in our analysis.

We employ an instrumental variable (IV) approach. Valid instruments enable us to uncover the causal effect of an endogenous explanatory variable on the dependent variable if, conditional on the other control variables, they are related to the endogenous explanatory variable (relevance condition) and unrelated to the dependent variable (exclusion restriction). Our IV strategy considers some (regional) institutional and political features that we use to instrument environmental technologies as exogenous drivers of regional GHG emissions.

The first instrument is a measure of regional institutional quality (*Quality of Institutions*), constructed at the NUTS 2 level. This variable is a composite indicator of institutional quality based on the EU Regional Social Progress Index and extended with other dimensions measuring the role played by public institutions in supporting inventive activities and R&D investment (Bianchini et al. 2019). The higher the value of this index, the better the definition and protection of a region’s intellectual property rights, and the better the delivery by the region’s institutions of public goods and services that can facilitate business and innovation activities leading to knowledge creation, innovation diffusion and absorption. Thus, the presence of institutions that support innovation activity by reducing the uncertainty inherent to any innovation project—including green innovations—should stimulate patent applications (our endogenous regressor) but not directly stimulate emissions (our dependent variable). Some studies have shown that institutional quality may contribute to abating emissions, especially at the national level (Dasgupta and de Cian 2018); however, here we use an indicator at the NUTS 2 level, a more granular level of administration that should not directly influence climate policies and global emissions. Moreover, our indicator is more fully focused on inventive and R&D activities and, as such, is more likely to be correlated with our explanatory variable (patents) than with our dependent variable (GHG emissions), as confirmed by pairwise correlation.

A second set of instruments is constructed at the NUTS 3 level and captures the *political orientation* of the region. Our choice is based on evidence that civil society can affect the political agenda (e.g., by influencing public R&D spending—Filippetti and Vezzani 2022) and that political orientation reflects, at least in part, local environmental awareness of that society. This, in turn, may generate stronger support for the creation of cleantech start-ups as well as stimulating policies targeted at sustaining inventive activities in the green realm, which generally require long-term economic planning and ad-hoc incentives (Giudici et al. 2019). Thus, we consider the right-left orientation (*RILE*) of the party winning the regional elections, measured by drawing on an approach outlined in the political science literature (Budge and Laver 1992) and proposed by the authors of the Manifesto Project Database (2020). In short, they scrutinised the political manifestos of all the parties participating in the national elections of the countries included in our sample and constructed a measure of right-left orientation by applying a composite indicator of the various dimensions reported in Table 7 in Appendix B.

The RILE indicator (*RILE*) takes positive values (0;100] when the party is right-oriented—i.e., it encourages an economic model based on free-market policies and *laissez-faire* capitalism—and it takes negative values [−100; 0) when the party is left-oriented—i.e., it speaks favourably of the need for the State to support the creation of a fair, open market economy and to implement the long-term economic planning deemed essential for innovation activities to flourish, and it supports a State that assumes the risks of innovation failure that these policy actions may generate. The expectation is that the more right-oriented a region is, the lower its innovation activities (including those within the environmental domain), and, hence, it is a good predictor of our endogenous variable. We expect the instrument to be uncorrelated with local emissions, as GHG emission standards are set at the national and EU policy levels. Indeed, unlike such pollutants as PM_2.5_ and PM_10_, NUTS 3 administrations have no direct authority to influence GHG emissions.

We regionalised the RILE indicator—available at the year-party-nation level—by drawing on the regional election database that contains information on NUTS 3 election results, following the approach discussed in Santoalha and Boschma (2021). In addition to the continuous regional RILE indicator, which measures the right-left orientation of the party obtaining most votes at local elections, we also constructed indicators measuring polarised party preferences by creating two dummy variables taking values equal to 1 if the party obtaining most votes at local elections belongs to the first 10th (*Extreme Left*) or the last 90th percentile (*Extreme Right*) of the RILE distribution. The rationale behind this discrete version of the RILE is that majority parties with an extreme policy orientation are more likely to break path dependence in local policymaking and, therefore, to satisfy the relevance condition.

## Results

This section shows and discusses the results of the econometric analysis. We discuss estimates of Tobit (in Sect. 4.1) and IV (in Sect. 4.2) models in terms of elasticities—i.e., the percentage change in GHG emissions implied by a 1% increase in green and digital patents.

### Tobit Estimates

Table 2 reports the coefficient estimates from Eq. (1) above. Model (1) only includes green patents and serves as a benchmark against previous research; Model (2) incorporates digital patents as well; and Model (3) is the full specification and contains the interaction effect between environmental and digital technologies.^14^

**Table 2:** Tobit estimates—the effect of green and digital technologies on regional emissions

	(1)	(2)	(3)
Green Tech. (log)	−1.026***	−0.989***	−0.957***
	(0.092)	(0.119)	(0.119)
Digital Tech. (log)		−0.146	1.007**
		(0.253)	(0.499)
Green × Digital Tech. (log)			−0.297***
			(0.094)
Population (log)	4.833***	4.837***	4.834***
	(0.134)	(0.134)	(0.135)
Value added Manuf. (log)	1.873***	1.871***	1.840***
	(0.214)	(0.214)	(0.214)
Dirty Units (log)	0.103**	0.103**	0.107**
	(0.046)	(0.046)	(0.046)
Intermediate	0.767***	0.762***	0.768***
	(0.220)	(0.221)	(0.220)
Rural	0.353	0.352	0.401
	(0.253)	(0.253)	(0.254)
Pre-sample Mean GHG (log)	0.481***	0.481***	0.480***
	(0.011)	(0.011)	(0.011)
Year Dummies	Yes	Yes	Yes
# Observations	10,510	10,510	10,510
Log Likelihood	−30,938.38	−30,938.26	−30,935.70
Wald Test	5829.67***	5829.68***	5835.37***

Robust standard errors in parentheses, clustered at metroregion-level: ***, **, *, indicate significance at the 1%, 5% and 10% level, respectively

Overall, technological knowledge seems to affect emissions. First, we find that the regional endowment of environmental technologies helps reduce emissions, thus confirming H1. All specifications point to a significant, beneficial influence (negative coefficient) of green technologies on GHG emissions, consistent with expectations (see literature review in Sect. 2.1.).

Second, in line with H2, the complete model specification, which includes the direct impact of digital technologies and the interaction term, shows that the opposite effect holds for the regional endowment of digital technologies, which seem to have a negative impact on the environment. Finally, the interaction between green and digital technologies presents a negative coefficient. In line with H3, our estimates confirm that the joint presence of regional capabilities in the spheres of both green and digital technologies are mutually reinforcing and contribute to a reduction in GHG emissions.

Our control variables conform to expectations. Population, the share of value added in manufacturing, and the prevalence of highly polluting units are positively associated with regional GHG emissions. Compared to urban centres, intermediate areas pollute more. Regions specialised in energy intensive sectors tend to pollute more. Finally, environmental performance is shown to be strongly path dependent, as the average level of emissions prior to the period under analysis has a marked influence on future emissions.

### Instrumental Variable Estimates

The results of the first stage regressions on the alternative specifications (see Table 8 in Appendix B) considering a different set of candidate instruments indicate that the instruments are valid and sufficiently strong. Specifically, metro-regions with higher institutional quality and with majority voting preferences for extreme-left (extreme-right) parties show a statistically significantly higher (lower) propensity to patenting in environmental technologies. The specification with *Quality of institutions* and *Extreme Left* also satisfies the test of over-identification restrictions, so our comments below address these results.

The IV estimates (Table 3) provide stronger support for the hypotheses developed in the conceptual framework of this study. Once again, we see that environmental technologies have a beneficial effect on GHG emissions (consistent with H1), digital technologies have a detrimental impact on GHG emissions (consistent with H2), and environmental and digital technologies positively interact in the reduction of GHG emissions (consistent with H3).

**Table 3:** IV Estimates—the effect of green and digital technologies on regional emissions

	(1)	(2)	(3)
Green Tech. (log) [Instrumented]	−1.102***	−2.178***	−2.220***
	(0.110)	(0.255)	(0.256)
Digital Tech. (log)		2.019***	2.887***
		(0.383)	(0.549)
Green [Instr.] × Digital Tech. (log)			−0.184***
			(0.070)
Population (log)	3.928***	4.113***	4.112***
	(0.104)	(0.112)	(0.112)
Value Added Manuf. (log)	1.347***	1.454***	1.445***
	(0.155)	(0.156)	(0.156)
Dirty Units (log)	0.054	0.050	0.052
	(0.033)	(0.033)	(0.033)
Intermediate	0.276	0.022	0.009
	(0.179)	(0.186)	(0.186)
Rural	−0.118	−0.500**	−0.497**
	(0.201)	(0.216)	(0.215)
Pre-sample Mean GHG (log)	0.390***	0.403***	0.403***
	(0.008)	(0.009)	(0.009)
Year Dummies	Yes	Yes	Yes
# Observations	10,510	10,510	10,510
Adjusted R^2^	0.399	0.401	0.401
Over-identification Test [χ^2^ (*p*-value)]	1.900	2.259	2.158
	(0.168)	(0.133)	(0.142)

First step estimates in Table 8. Robust standard errors in parentheses, clustered at metroregion-level: ***, **, *, indicate significance at the 1%, 5% and 10% level, respectively

Point estimates suggest a fall (rise) in GHG emissions of about 2.20% (2.90%) in response to a 1% increase in environmental (digital) patents, while a 1% increase in both environmental and digital patents leads to an additional reduction of about 0.20%. While we should treat the magnitude of these estimates with caution, the point estimates associated with both environmental and digital technologies become stronger when accounting for endogeneity.

### Model Implications

The logic underpinning the “twin” transition, endorsed by recent policy efforts, seems to find only partial support in our analysis. In fact, the net effect of a joint increase in digital and environmental technologies on GHG emissions is, on average, negative, given that the benefits that accrue from the combination of the direct effect of environmental technologies and the interaction of these with digital technologies, only partly offset the detrimental impact of the direct effect of digital technologies. However, a more detailed analysis is required if we hope to derive clear policy implications. This involves considering the joint distribution of environmental and digital technology endowments across metro-regions, on the understanding that their effects vary with their respective endowments.

**Fig. 5:**
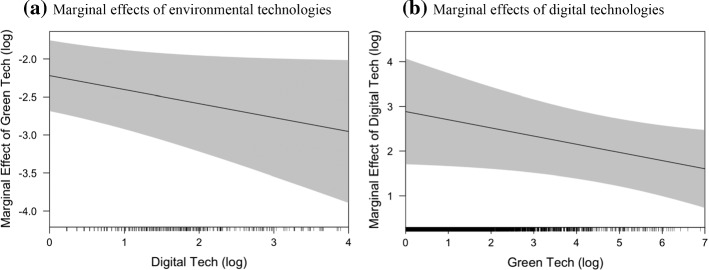
Marginal effects of environmental and digital technologies on GHG emissions. *Notes*: Marginal effects are computed using point estimates of the IV model 3 of Table 3. The shaded area represents the 95% confidence intervals

Figure 5 shows the marginal effects of environmental (digital) technologies on GHG emissions across different levels of digital (environmental) technologies. Panel (a) shows the negative and statistically significant marginal effects of environmental technologies (i.e., beneficial for GHG reduction) across the whole domain of digital technologies (horizonal axis). The negative slope of the marginal effects indicates that the beneficial effect of environmental technologies on emissions increases with the endowment of digital technology capabilities. Panel (b) shows the positive and significant effect of digital technologies on GHG emissions (i.e., detrimental for GHG emissions), which also decreases as the regional endowment of environmental technology capabilities increases.

**Fig. 6:**
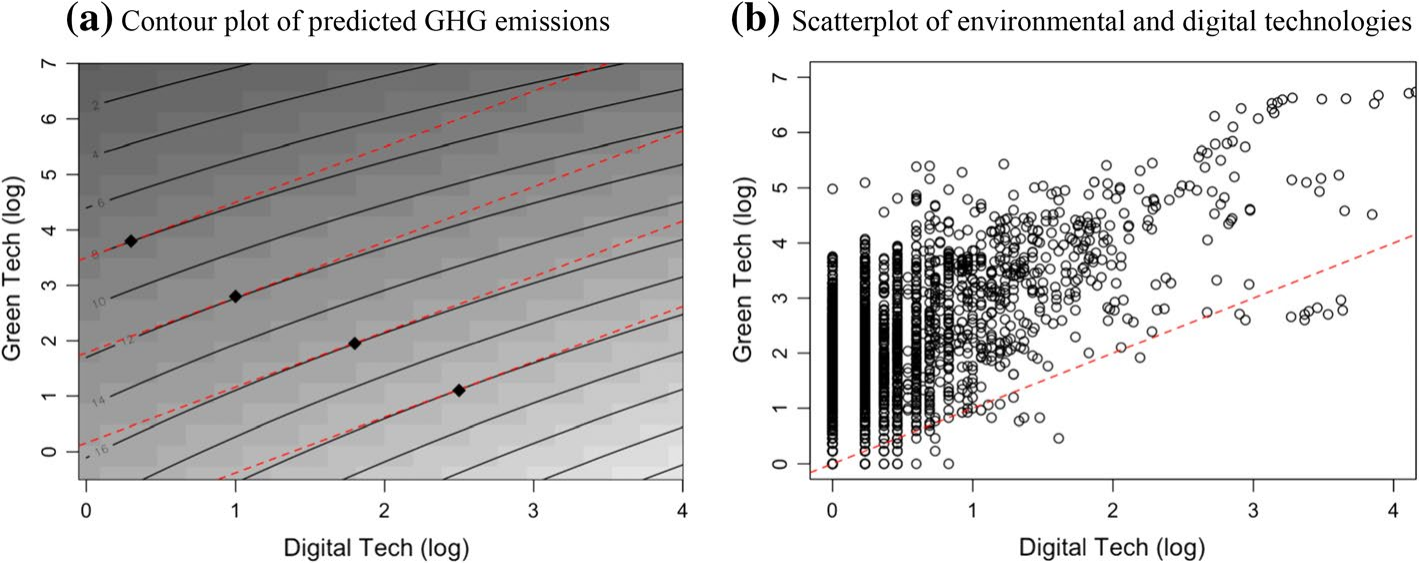
Visualisation of the main effects of the “twin” transition. *Notes*: The contour plots are computed using point estimates of the IV model 3 of Table 3

To understand the combined implications of these effects, it is informative to consider the contour plot of *predicted* GHG emissions at varying levels of environmental and digital technology capabilities (Fig. 6, panel a), together with the joint distribution of environmental and digital technologies across metro-regions (Fig. 6, panel b). Indeed, Fig. 6 provides a clear interpretation of the mix of synergies and trade-offs at work here. Specifically, the contour lines in panel (a) indicate combinations of environmental and digital technologies associated with the same GHG emission levels, with lower GHG emission levels being associated with higher environmental and lower digital endowments (top-left of the graph). When the slope of the curve is equal to one, a (marginal) joint “twin” (i.e., of similar magnitude) increase in environmental and digital technology endowments would leave emissions unchanged. The locus of the tangency points of the contour lines and the parallel lines of the bisector (henceforth, “locus”, for the sake of brevity) contain all the combinations that satisfy such a condition: the locus can be imagined as a line cutting across the plot from the north-west to the south-east. The combinations of technology endowments whose contour line has a slope larger (smaller) than one—i.e., those to the left (right) of the locus—require larger (smaller) increases in environmental technologies than in digital technologies to maintain the same level of GHG emissions.

The joint distribution of environmental and digital technologies across metro-regions plotted in panel (b) shows that most region-year observations in the sample lie to the left of the locus. More than 80% of the sample shows a combination of endowments lower than 2 (log) environmental technologies and 1 (log) digital technologies. This implies that more than half of Europe’s metro-regions would experience an increase in GHG emissions in the case of a “twin”—equal in magnitude—increase in environmental and digital technology endowments. In contrast, a disproportionately larger increase in environmental technologies is required to maintain or reduce GHG emissions. Only in the case of the very few macro-regions with medium-large environmental and digital endowments lying to the right of the locus (c. 1% of the sample with a combination of endowments larger than 3 (log) environmental technologies and 2 (log) digital technologies) would an equal increase in environmental and digital technology endowments reduce the level of GHG emissions.

### Unbounding the Digital Ecosystem

We have, up to this juncture, considered the digital ecosystem as a set of interconnected technologies that interoperate with and complement one another. However, it should prove insightful to extend this analysis by seeking to understand the heterogeneous effects of the different components of the ecosystem on the GHG emissions from production activities.

While we do not have any specific ex-ante expectations regarding differences in impact, previous efforts in this direction—namely, Dusik et al. (2018) and Bond and Dusík (2020)—have considered a wide array of environmental and social implications of four digital components of the digital ecosystem: that is, additive manufacturing, AI, IoT and robotics. According to these studies, additive manufacturing and AI can be expected to have the most beneficial effects in the abatement of GHG emissions, with a best-case scenario rated as ‘moderately positive’ and a worst-case scenario as ‘moderately adverse’. Advanced industrial robotics occupies an intermediate position, with projected scenarios ranging from ‘neutral’ to ‘significantly adverse’, while IoT appears to have the most damaging impact, with scenarios ranging from ‘moderately adverse’ and ‘significantly adverse’ for GHG emissions.

In the context of this study, we can safely assume that the main benefits for GHG emissions originating from additive manufacturing and AI lie in their potential reduction of energy, attributable to customised production processes in the case of the former and to system optimisation and a better management of energy use in the case of the latter. These potential energy savings may, however, be offset by the energy requirements to produce increasing quantities of products in the case of additive manufacturing and the interconnection of the production system in the case of AI. The comparatively gloomier scenarios assumed for robotics and IoT reflect expectations of a significantly increased demand for electricity and the digital waste created by the proliferation of electronic appliances and equipment.

The Tobit estimates—broken down into the six categories that make up our digital ecosystem—provide some support for the above conjectures (Table 4). Specifically, Robotics and, in particular, Additive Manufacturing have a significant direct detrimental effect (i.e., presenting a positive coefficient) on GHG emissions, while AI and IoT have no statistically significant effects. Additionally, we found particularly strong statistically significant detrimental effects of Big Data and Computing Infrastructures, reflecting possibly their high energy requirements and low expectations of any positive environmental returns. The interaction terms are negative and statistically significant for most of the components (with the sole exception of AI where the negative effect is not statistically significant), but are of a smaller magnitude than the direct effects, while the differences are smaller across components.

**Table 4:** Tobit estimates—the effect of the individual digital technology categories on regional emissions

	(1)	(2)	(3)	(4)	(5)	(6)
Green Tech (log)	−1.013***	−0.890***	−0.994***	−0.974***	−0.943***	−0.977***
	(0.097)	(0.106)	(0.093)	(0.094)	(0.100)	(0.111)
Additive Manuf. (log)	2.144*					
	(1.185)					
Green × Additive Manuf. (log)	−0.531**					
	(0.216)					
AI (log)		−0.787				
		(1.222)				
Green × AI (log)		−0.143				
		(0.207)				
Big Data (log)			5.456*			
			(3.294)			
Green × Big Data (log)			−1.474***			
			(0.535)			
Computing Infra. (log)				6.158**		
				(2.802)		
Green × Computing Infra. (log)				−1.603***		
				(0.449)		
IoT (log)					2.100	
					(1.938)	
Green × IoT (log)					−0.662**	
					(0.323)	
Robotics						1.349**
						(0.603)
Green × Robotics (log)						−0.377***
						(0.116)
Population (log)	4.832***	4.848***	4.834***	4.841***	4.840***	4.836***
	(0.134)	(0.135)	(0.135)	(0.134)	(0.134)	(0.134)
Value Added Manuf. (log)	1.866***	1.853***	1.858***	1.849***	1.848***	1.837***
	(0.214)	(0.214)	(0.214)	(0.214)	(0.214)	(0.215)
Dirty Units (log)	0.106**	0.107**	0.105**	0.106**	0.107**	0.107**
	(0.046)	(0.046)	(0.046)	(0.046)	(0.046)	(0.046)
Intermediate	0.771***	0.747***	0.783***	0.774***	0.772***	0.769***
	(0.220)	(0.220)	(0.220)	(0.220)	(0.220)	(0.220)
Rural	0.371	0.370	0.380	0.387	0.390	0.395
	(0.253)	(0.253)	(0.253)	(0.253)	(0.253)	(0.253)
Pre-sample Mean GHG (log)	0.481***	0.479***	0.481***	0.481***	0.480***	0.480***
	(0.011)	(0.011)	(0.011)	(0.011)	(0.011)	(0.011)
Year Dummies	Yes	Yes	Yes	Yes	Yes	Yes
# Observations	10,510	10,510	10,510	10,510	10,510	10,510
Log-likelihood	−30,937.39	−30,935.07	−30,936.41	−30,934.44	−30,935.33	−30,935.82
Wald Test	5831.87***	5837.37***	5834.74***	5839.66***	5837.26***	5835.03***

Robust standard errors in parentheses, clustered at metroregion-level: ***, **, *, indicate significance at the 1%, 5% and 10% level, respectively

Taken together, these findings suggest that the detrimental impact of the whole ecosystem on GHG emissions is mostly driven by specific digital technologies. It goes without saying that further research is needed to consolidate these results.

## Conclusion

The possibilities afforded by advanced digital technologies have been met with equal doses of enthusiasm and trepidation and this has much to do with the challenges posed by the climate crisis. The European Union is making an unprecedented effort to promote a “twin” green and digital transition aimed at creating a more sustainable, fairer, and prosperous society. Yet, the doubt remains as to whether a green and a digital transition constitute a winning pair for the environment or whether one transition risks inhibiting the other. This study has examined these two questions both theoretically and empirically and offers the following main findings.

First, a considerable degree of technological disparity still reigns in the European landscape. Our newly constructed dataset indicates that many regions in Europe, especially the East and the periphery, lag behind in terms of their digital technology development. This is not, however, the case with green technologies, which perhaps find themselves at a more advanced stage of their life cycle. Second, our findings cast some doubt on the effectiveness of the “twin” transition in supporting GHG emissions *tout court*. While the local development of green technologies reduces GHG emissions, the local development of digital technologies has a negative effect on the environment, which is only partially mitigated in regions that are sufficiently endowed with green technological knowledge. And, third, it seems that not all components of the digital ecosystem can be held equally responsible for this negative impact on the environment, because when we unbundle the components, our results seem to suggest that the most energy intensive elements have the most detrimental effect. Taken together, these findings highlight the importance of strategically tailoring any “twin” transition policy to the technological capacity of Europe’s regions, because stimulating digital transformation by means of a “one-size-fits-all” approach may have severe consequences for the environment of targeted regions.

While these are the main conclusions to be drawn from this study, additional considerations and implications also emerge. Having established that the digital transition poses severe environmental challenges, the response cannot be simply to abandon these technologies altogether, but rather to recognise both their potential and their limitations. Government policy has the power to direct technological progress towards achieving certain goals, including environmental targets, and this applies also to digital technologies. In this regard, it is not only “hard” policy instruments—that is, stricter regulation of the direct and indirect effects of digital technologies, for example, of their energy uses and the disposal of certain components—but also “soft” instruments that are needed. Users, for instance, need to be better informed about what the consumption of digital goods and services actually entails. Both individual and institutional initiatives should be triggered to systematically track the environmental costs of digital transformation and so raise awareness among digital practitioners. This would go some way to countering the somewhat overly optimistic faith currently placed in digitalisation by the press and popular science with their almost unique focus on the successes of digital technologies. Second, other metrics of technological success could be adopted when evaluating innovation, allowing users to internalise measures of their environmental footprint–such as energy used per unit of production, emissions released, and environmental degradation—and these metrics should also become guidelines for the establishment of broader ethical principles (Coeckelberg 2021b), something that has already been suggested for certain technologies. For example, Strubell et al. (2019) highlight the importance of quantifying the financial and environmental costs of training deep learning models for NLP tasks. In the future, this reasoning can usefully be extended to other digital technologies.

This study is not without its limitations. First, our sample only includes the industrial emissions of highly energy-intensive and highly polluting plants, overlooking altogether industrial emissions from less intensive plants. Plants subject to the European Trading Scheme are responsible for c. 45% of GHG emissions, which means our sample excludes more than half of all emissions.

Second, it assumes that those highly polluting plants use electricity generated in their metropolitan area. To avoid network losses in the grid due to transmission we can reasonably exclude that most of the electricity consumed in a region is produced very far away. Yet, we cannot rule out the possibility that it may come from neighbouring provinces. However, most metro-regions are likely to be equipped in their territory of thermo-electric power plants, that will serve their electricity needs, thus limiting the consequences of the bias. Data for Italy show, for example, that only 7% of Italian provinces have no thermo-electric power plants on their territory and that there is a positive correlation between the number of plants subject to reporting emission data and the installed capacity of thermo-electric power plants (measured in Kw/h). Provinces with many highly polluting plants are also those experiencing high production of electricity from thermo-electric non-renewable sources (mostly oil, methane and partly coke). Relatedly, it assumes the electricity use of those highly polluting plants is associated to resources that cause GHG emissions, namely excluding renewable energy sources. On the one side, those plants are likely to produce on a continuous process, making the seasonality and uncertainty in renewables a limit to their direct use for production. On the other side, we cannot fully exclude their energy use will include electricity that enters the grid being generated by renewables, especially during the day. This is a limitation that we could not solve.

Third, regional green and digital transitions are gauged in terms of patenting activity, which is used to measure the level of local technological knowledge. However, measuring innovations via the number of patent applications may underrepresent the innovation activities of certain firms that tend to rely less on patents to protect their inventions and overrepresent those sectors in which there is a tendency to register more patents. Moreover, as well as not being a perfect measure of innovations, the registering of a patent does not necessarily imply the local diffusion of that technology into the production ecosystem. Given the well-known limitations of data availability to measure technological adoption, we resume to proxy it through technological development by assuming that local spillovers are a key channel for the local diffusion of technological knowledge, supported by many previous studies documenting the important role of technological local spillovers. Results of our study can be extended by considering the role of spatial interconnections among regions in shaping regional environmental performance, further exploiting the potential of the original dataset that we have created. This can provide additional useful insights for policies related to the twin transition, in particular with respect to coordination mechanisms to be designed by supra-regional authorities (e.g., national and EU institutions) to improve the design of adequate instruments and the allocation of funding.

Fourth, our analysis has assessed the net aggregate effect of the joint digital and green transition on air emissions. While we use the metropolitan level of analysis as the most granular functional geographic resolution, case studies and quantitative analyses at the firm-level would provide further insights into how digital and green technologies combine and the internal mechanisms behind these combinations operate. To make such kinds of analysis possible, future research may exploit expert opinions and survey data focused on digital and green innovative activities at the firm-level.

Last, but certainly not least in terms of its relevance, the current study offers evidence for the European regions. Clearly, not all societies are equally vulnerable to the risks induced by the digital transition, and developing and less developed countries seem more likely to experience its downside. This points to the pressing need for new research in these areas to better quantify and qualify the environmental returns of the “twin” transition and also to the need to strengthen both digital *and* climate governance mechanisms at the global level.

## Figures and Tables

**Fig. 7:**
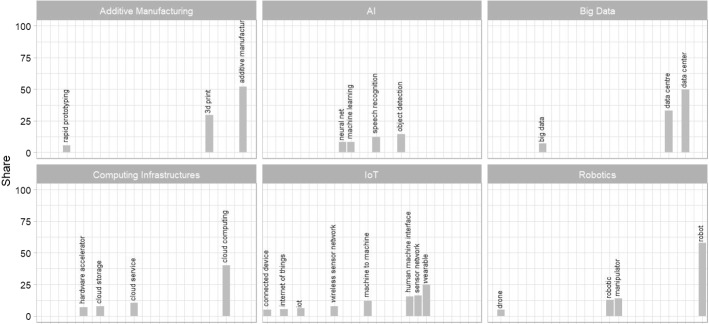
Most recurrent search terms, per category. *Notes*: Search terms are identified in the titles and abstract of digital patents. The figure shows only those terms with a share higher than 5%. They account for 81.66% of the total (i.e., roughly 82% of the patents can be assigned to the various categories with only 26 terms). For each category: 87.50% (Additive Manufacturing); 43.21% (AI) 89.93% (Big Data); 65.07% (Computing Infrastructure); 93.54% (IoT); 90.04% (Robotics)

**Fig. 8:**
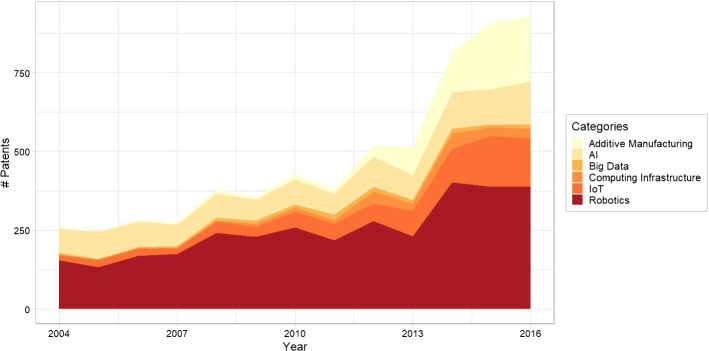
Trends in the number of digital patents, per category

**Fig. 9:**
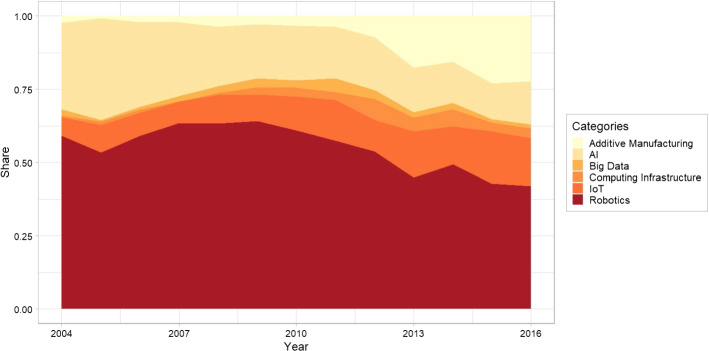
Trends in the share of digital patents, per category

## Appendix A: The digital ecosystem

This Appendix provides more details about the macro-components of the digital ecosystem. First, we describe each component. Second, we characterise the patent content of each component by showing the most recurrent keywords in the identified patent applications. Third, we briefly discuss the evolution of components absolute and relative size across time.

### Digital components

#### Additive Manufacturing

Additive manufacturing, or 3D printing, consists in the computer-controlled production of three dimensional objects by depositing materials, usually in layers, with precise geometric shapes. A technique for rapid prototyping system using photopolymer layers was proposed in 1981 by Hideo Kodama (Nagoya Municipal Industrial Research Institute). Soon afterwards it became possible to create complex models with the help of computer aided manufacturing or computer-aided design (CAM/CAD) software. The procedure came to be known as stereolithography: a liquid resin material is polymerised with a high-precision laser to form each layer, and the process is said to be “additive” because the objects are built layer by layer. The first 3D printing machines turned into a viable commercial product only in the early 2000s, and this paved the way for the production of industrial parts on demand.

Today, there are distinct AM processes, each with specific standards (details are largely beyond the scope of this document): vat polymerisation, material jetting, binder jetting, directed energy deposition, material extrusion, powder bed fusion, and sheet lamination. What characterise these processes is that, unlike traditional manufacturing, they do not require machining or other techniques to remove surplus material. The objects produced can achieve much finer details and the production process is more reliable as it can repeatedly achieve high quality results. 3D printed products can serve a variety of different applications ranging from automotive, healthcare, aerospace and parts replacement.

#### Artificial Intelligence

Human-like machines are described in many stories and are pictured in sculptures, painting, and drawings already from the Ancient Greeks. Long debate about what might be needed to make machines intelligent are scattered abundantly throughout philosophy, logic, biology, statistics, and engineering from the sixteenth century, reaching a peak in the mid-twentieth century with several breakthroughs in computation theory by the English logician and mathematician Alan Turing and the American mathematician, physician, and polymath John von Neumann.

The emergence of artificial intelligence as a full-fledged field of research coincides with three important meetings: Session on Learning Machines in 1955 (Los Angeles); Summer Research Project on Artificial Intelligence in 1956 (Dartmouth); and Mechanization of Thought Processes in 1958 (UK). The 1956 workshop is considered to be the official beginning of AI, whose overarching goal would have been to “*make machines use language, form abstractions and concepts, solve kinds of problems now reserved for humans, and improve themselves*” (McCarthy, Minsky, Rochester, and Shannon 1956).

Today, AI brings together a number of distinct and often intersecting sub-fields such as machine learning, computer vision, natural language processing, symbolic reasoning, knowledge representation, and many others. Recent definitions aim to be understandable, technically accurate, technology-neutral and applicable to short- and long-term horizons. Here are some examples: “*Machines or agents that are capable of observing their environment, learning, and based on the knowledge and experience gained, taking intelligent action or proposing decisions*” (EC 2018); “*An AI system is a machine-based system that can, for a given set of human-defined objectives, make predictions, recommendations or decisions influencing real or virtual environments*” (OECD 2019); “*Machines that can become better at a task typically performed by humans with limited or no human intervention*” (WIPO 2019).

Although drawing a precise boundary to artificial intelligence is an ongoing subject of debate, there is a general consensus on the methodological building blocks needed to mechanise human intelligence (Russel and Norvig 2020). These AI building blocks typically include four elements: *machine learning*, *natural language processing* (NLP), *computer vision* and *speech recognition*.^15^

As for machine learning, for instance, we include terms such as “neural network”, “deep learning” and “support vector machines”, which are essentially techniques for predictive analytics. NLP includes terms such as “knowledge representation”, “semantic search” and “sentiment analysis”. Computer vision comprehends, among others, terms such as “image classification”, “object detection” and “pose estimation”. And speech recognition includes, for example, “speech recognition” and “voice recognition”.

#### Big Data

The term was popularised by computer scientist John Mashey in the 1990s, referring to unusually large and heterogeneous data sets that were difficult to capture and process with commonly used software. More accurate definitions appeared in the early 2000s: “*Big data is high volume, high velocity, and/or high variety information assets that require new forms of processing to enable enhanced decision making, insight discovery and process optimisation*” (Douglas Laney 2001).

Today, much the same as for AI, there is no clear consensus on what is Big Data. Definitions often include (at least) three features, commonly referred to as the “3Vs of Big Data”. These are *Volume* or very large size; *Velocity* corresponding to the speed of data creation which should be in real-time or nearly-real time; and *Variety* representing the heterogeneity of data sources (e.g., text from messages, images posted to social networks, readings from sensors). Other Vs are added from time to time, such as *Veracity* (data quality), *Value* (value obtained from exploitation), and *Variability* (rate of change).

For instance, De Mauro et al. (2015) propose that Big Data can be considered as a standalone term referring to those “*Information assets characterized by such a High Volume, Velocity and Variety to require specific Technology and Analytical Methods for its transformation into Value*”, and as an attribute when denoting its peculiar requisites (e.g., Big Data Technology or Big Data Analytics).

The terms in our list adhere to this idea of autonomous terms with respect to standalone term *vis-à-vis* attributes. Indeed, we include terms such as “massive data” and “large-scale data” but also technological requisites such as “data centre” and “Hadoop”.

#### Computing Infrastructures

We refer to computing infrastructures as physical and virtual resources that support the flow, storage, processing and analysis of data. An infrastructure can either be centralised within a data center, or it can be decentralised and distributed in several data centers.

Algorithmic advances and the advent of Big Data have changed the way infrastructures are designed and implemented. For instance, demanding users rely more and more often on cloud computing for the provision of flexible on-demand computing services such as storage and processing.

More specifically, cloud computing encompasses the delivery of computing services – servers, storage, databases, networking, software, and analytics – over the Internet (i.e., the “cloud”). Cloud manufacturing embraces the application in manufacturing of cloud technologies, with widespread access, easy and on-demand IT services to support production processes and supply chain management. The concept of infrastructure-as-a-service (IaaS) dates back to the 1960s but became fully operational for users in the early 2000s. Recent technologies such as fog computing and 5G extends the benefits of IaaS, providing a far higher level of performance (high speed and low latency) than the previous generations of computing and mobile communications systems. Furthermore, computational capabilities have experienced a tremendous increase in the past decades, and this has been made possible by new computational approaches (many of which are still in an experimental phase) such as quantum computing.

We include terms referring to cloud computing, such as “cloud architecture” and “on-demand computing”, but also terms referring to computing power, such as “hardware accelerator” and “supercomputing”.

#### Internet of Things

The idea of connecting a physical object to the Internet dates to 1982, when a Coke machine was first connected to the Internet at Carnegie Mellon University. In the early 1990s, the idea of a physical connection to the Internet became more and more pervasive.

Today, IoT is a concept describing a wide ecosystem of interconnected devices and services that collect, exchange and process data to adapt dynamically to a given context (Atzori et al. 2010). IoT entails networks of physical objects (the “things”) embedded with ambient sensors and dedicated software, and connected via standard communication protocols.

The underlying technologies needed to build an IoT device are semiconductor technologies, sensor technologies and more generally micro-electromechanical systems (MEMS), and of course the Internet. When connected to each other, the network of “things” offers self-identification, localisation, diagnostic status, data acquisition and processing capabilities. Data and information can moreover be collected from a wide variety of sources (industrial products, transport vehicles, etc.). IoT allows objects to interact with other objects and therefore with people in an increasingly digitalised and automatized way.

For IoT, we are also faced with a high degree of technological complexity. Among the terms on our list, we include sensor-related technologies such as “pervasive sensing” and “smart sensor”, and other technologies referring to means of communication, such as “hyper connectivity”. We consider both applications in industry (e.g., “machine-to-enterprise” and “smart factor”) and home automation and domestic appliances (e.g., “connected home” and “smart home”).

#### Robotics

Robotics encompasses agents with different capabilities to substitute for humans and replicate and automate human actions. Although robotics has a long and rich history, with inventions that intertwined in various scientific domains—information and mechanical engineering, computer science, etc.—and visionary insights, the first commercial robots installed for industrial purposes appeared in the 1960s. But it was only after the 1980s that we witnessed a massive deployment of (multitasking) industrial robots aimed at automatizing the mass production of consumers and industrial goods.

Modern flexible robots, empowered by machine learning systems, can interact with the environment, self-learn from the environment and improve with experience. Robots find applications in various segments of the economy: manufacturing, assembly and packing, transportation, earth and space exploration, surgery and patient healthcare, laboratory R&D, but also household chores.

Industrial robots are often classified in various subgroups, depending on their anthropomorphic characteristics, the type of movements they can perform and the plane of action (e.g., horizontal, vertical, rotary). Among these groups, we typically find SCARA (Selective Compliance Assembly Robot Arm), articulated, Cartesian, dual arm robots and cobots (Nilsson 2009; Russel and Norvig 2020).

Our terms are broad in scope and allow to identify physical components of a robot (e.g., “robotic” arm/leg/fingers), some of its functionalities (e.g., “manipulator”), and control systems (e.g., “robot” control/plan/movement). We also consider an increasingly active area of research dealing with autonomous vehicles in various environments such as land, water, and air.

### Most recurrent keywords

Figure 7 shows the most recurring search terms in the titles and abstracts of digital patents. It makes evident the overrepresentation of a few terms, typically closely related to the general scope of the category – e.g., “additive manufacturing” or “3d print”. AI and IoT are an exception in that the recurring terms are more heterogeneous and refer to data analysis techniques (e.g., “neural network”), AI applications (“object detection”), and communication systems (“machine-to-machine”).

### Time changes of the size of the components of the digital ecosystem

Figure 8 shows the evolution of patenting activity in the digital realm. We see a steady increase since the early 2000s, with the number of patents soaring since 2013. Robotics is the most representative category, counting a total of 3266 patents throughout the period, followed by AI (1139); IoT (808); Additive Manufacturing (743); Computing Infrastructures (211); and Big Data (129). The acceleration from 2010s can be partially explained as the response of the scientific system to some breakthroughs in multilayer neural networks. These discoveries seem to have brought about a hype toward digital technology of all kinds.

Patenting activity has become particularly dynamic for some technologies, as shown in Fig. 9. For example, the share of patents related to Additive Manufacturing at the beginning of the period was close to zero but reached about 25% in 2016. Similar trends have occurred for IoT. On the contrary, patenting in Robotics and Artificial Intelligence, while growing at a high pace, have lost some ground in relative numbers. The contribution of patents associated with Big Data and Computing Infrastructure is always marginal, even in the most recent period.

## Appendix B: Additional statistics

See Tables

**Table 5:** Descriptive statistics

	Mean	SD	Min	25° Perc	75° Perc	Max
*Main variables*						
GHG Emission (log)	14.447	8.760	0	8.974	21.072	25.211
Pre-sample Mean GHG (log)	11.869	9.348	0	0	20.125	25.147
Green Tech. (no. patents)	6.016	34.829	0	0	3	858
Digital Tech. (no. patents)	0.475	3.369	0	0	0	129
*Digital ecosystem*						
Additive Manufacturing	0.061	0.764	0	0	0	44
AI	0.079	0.628	0	0	0	18
Big Data	0.011	0.163	0	0	0	9
Computing Infrastructures	0.018	0.290	0	0	0	14
IoT	0.063	0.763	0	0	0	35
Robotics	0.240	1.752	0	0	0	71
*Controls*						
Population	471,063	787,182	19,504	148,536	528,340	13,998,563
Value Added Manufacturing	23.870	10.454	1.817	30.214	30.214	73.563
Dirty Units	2499	4573.926	0	2876	2876	112,496
Urban	0.203	0.402	0	0	0	1
Intermediate	0.409	0.492	0	1	1	1
Rural	0.388	0.487	0	1	1	1
*Instruments*						
RILE	−3.277	16.257	−93.490	0	0	56.040
Extreme Left	0.093	0.291	0	0	0	1
Quality of Institutions	58.080	13.323	20.499	48.755	67.645	86.354

Statistics refer to the sample for the econometric analysis (2007–2016)—10,510 observations

5,

**Table 6:** Pairwise correlations

	1	2	3	4	5	6	7	8	9	10	11	12	13	14
1. GHG Emissions (log)	1	0.526	0.274	0.353	0.184	0.495	0.079	0.174	0.202	0.032	−0.198	−0.042	0.061	−0.009
2. Pre-sample Mean GHG (log)	0.526	1	0.331	0.405	0.188	0.344	0.072	0.152	0.197	0.003	−0.165	−0.066	0.098	0.234
3. Green Tech. (log)	0.274	0.331	1	0.818	0.689	0.554	0.0005	0.168	0.437	−0.049	−0.311	−0.013	0.048	0.372
4. Green Tech. (log) [Instrumented]	0.353	0.405	0.818	1	0.843	0.677	0.001	0.206	0.535	−0.060	−0.381	−0.018	0.059	0.455
5. Digital Tech. (log)	0.184	0.188	0.689	0.843	1	0.425	−0.027	0.135	0.338	−0.073	−0.205	−0.010	0.034	0.188
6. Population (log)	0.495	0.344	0.554	0.677	0.425	1	−0.051	0.276	0.454	−0.003	−0.371	0.006	0.072	−0.097
7. Value Added Manufacturing (log)	0.079	0.072	0.0005	0.001	−0.027	−0.051	1	0.050	−0.179	0.084	0.063	0.056	−0.133	0.157
8. Dirty Units (log)	0.174	0.152	0.168	0.206	0.135	0.276	0.050	1	0.139	−0.026	−0.088	0.026	0.081	−0.006
9. Urban	0.202	0.197	0.437	0.535	0.338	0.454	−0.179	0.139	1	−0.420	−0.402	0.086	−0.034	0.096
10. Intermediate	0.032	0.003	−0.049	−0.060	−0.073	−0.003	0.084	−0.026	−0.420	1	−0.663	0.043	−0.053	−0.046
11. Rural	−0.198	−0.165	−0.311	−0.381	−0.205	−0.371	0.063	−0.088	−0.402	−0.663	1	−0.114	0.081	−0.033
12. RILE	−0.042	−0.066	−0.013	−0.018	−0.010	0.006	0.056	0.026	0.086	0.043	−0.114	1	−0.714	−0.045
13. Extreme Left	0.061	0.098	0.048	0.059	0.034	0.072	−0.133	0.081	−0.034	−0.053	0.081	−0.714	1	−0.009
14. Quality of Institutions (log)	−0.009	0.234	0.372	0.455	0.188	−0.097	0.157	−0.006	0.096	−0.046	−0.033	−0.045	−0.009	1

^6^,

**Table 7:** List of dimensions considered to construct the composite indicator on right-left orientation RILE (used in the IV strategy)

Dimensions that positively contribute to RILE index (right-oriented policy manifesto)	Dimensions that negatively contribute to RILE index (left-oriented policy manifesto)
**Military: positive** The importance of external security and defence. May include statements concerning: • The need to maintain or increase military expenditure; • The need to secure adequate manpower in the military; • The need to modernise armed forces and improve military; • Strength; • The need for rearmament and self-defence; • The need to keep military treaty obligations **Freedom and human rights** Favourable mentions of importance of personal freedom and civil rights in the manifesto and other countries. May include mentions of: • The right to the freedom of speech, press, assembly etc.; • Freedom from state coercion in the political and economic spheres; • Freedom from bureaucratic control; • The idea of individualism **Constitutionalism: positive** Support for maintaining the status quo of the constitution. Support for specific aspects of the manifesto country’s constitution. The use of constitutionalism as an argument for any policy **Political authority** References to the manifesto party’s competence to govern and/or other party’s lack of such competence. Also includes favourable mentions of the desirability of a strong and/or stable government in general **Free market economy** Favourable mentions of the free market and free market capitalism as an economic model. May include favourable references to: • Laissez-faire economy; • Superiority of individual enterprise over state and control systems; • Private property rights; • Personal enterprise and initiative; • Need for unhampered individual enterprises **Incentives: Positive** Favourable mentions of supply side oriented economic policies (assistance to businesses rather than consumers). May include: • Financial and other incentives such as subsidies, tax breaks etc.; • Wage and tax policies to induce enterprise; • Encouragement to start enterprises **Protectionism: negative** Support for the concept of free trade and open markets. Call for abolishing all means of market protection (in the manifesto or any other country) **Economic Orthodoxy** Need for economically healthy government policy making. May include calls for: • Reduction of budget deficits; • Retrenchment in crisis; • Thrift and savings in the face of economic hardship; • Support for traditional economic institutions such as stock market • and banking system; • Support for strong currency	**Anti-imperialism** Negative references to imperial behaviour and/or negative references to one state exerting strong influence (political, military or commercial) over other states. May also include: • Negative references to controlling other countries as if they were part of an empire; • Favourable references to greater self-government and independence for colonies; • Favourable mentions of de-colonisation **Military: negative** Negative references to the military or use of military power to solve conflicts. References to the ‘evils of war’. May include references to: • Decreasing military expenditures; • Disarmament; • Reduced or abolished conscription **Peace** Any declaration of belief in peace and peaceful means of solving crises– absent reference to the military. May include: • Peace as a general goal; • Desirability of countries joining in negotiations with hostile countries; • Ending wars in order to establish peace **Internationalism: Positive** Need for international co-operation. May also include references to the: • Need for aid to developing countries; • Need for world planning of resources; • Support for global governance; • Need for international courts; • Support for UN or other international organisations **Democracy** Favourable mentions of democracy as the “only game in town”. General support for the manifesto country’s democracy. May also include: • Democracy as method or goal in national, international or other organisations (e.g. labour unions, political parties etc.); • The need for the involvement of all citizens in political decisionmaking; • Support for either direct or representative democracy; • Support for parts of democratic regimes (rule of law, division of powers, independence of courts etc.) **Market regulation** Support for policies designed to create a fair and open economic market May include: • Calls for increased consumer protection; • Increasing economic competition by preventing monopolies and other actions disrupting the functioning of the market; • Defence of small businesses against disruptive powers of big businesses; • Social market economy **Economic Planning** Favourable mentions of long-standing economic planning by the government. May be: • Policy plans, strategies, policy patterns etc.; • Of a consultative or indicative nature
**Welfare state limitation** Limiting state expenditures on social services or social security. Favourable mentions of the social subsidiary principle (i.e. private care before state care); **National way of life: positive** Favourable mentions of the manifesto country’s nation, history, and general appeals. May include: • Support for established national ideas; • General appeals to pride of citizenship; • Appeals to patriotism; • Appeals to nationalism; • Suspension of some freedoms in order to protect the state against subversion **Traditional morality: positive** Favourable mentions of traditional and/or religious moral values. May include: • Prohibition, censorship and suppression of immorality and unseemly behaviour; • Maintenance and stability of the traditional family as a value; • Support for the role of religious institutions in state and society **Law and Order: Positive** Favourable mentions of strict law enforcement, and tougher actions against domestic crime. Only refers to the enforcement of the status quo of the manifesto country’s law code. May include: • Increasing support and resources for the police; • Tougher attitudes in courts; • Importance of internal security **Civic mindedness: positive** Appeals for national solidarity and the need for society to see itself as united. Calls for solidarity with and help for fellow people, familiar and unfamiliar. May include: • Favourable mention of the civil society; • Decrying anti-social attitudes in times of crisis; • Appeal for public spiritedness; • Support for the public interest	**Protectionism: Positive** Favourable mentions of extending or maintaining the protection of internal markets (by the manifesto or other countries). Measures may include: • Tariffs; • Quota restrictions; • Export subsidies **Controlled economy** Support for direct government control of economy. May include, for instance: • Control over prices; • Introduction of minimum wages **Nationalisation** Favourable mentions of government ownership of industries, either partial or complete; calls for keeping nationalised industries in state hand or nationalising currently private industries. May also include favourable mentions of government ownership of land **Welfare State Expansion** Favourable mentions of need to introduce, maintain or expand any public social service or social security scheme. This includes, for example, government funding of: • Health care; • Child care; • Elder care and pensions; • Social housing **Education expansion** Need to expand and/or improve educational provision at all levels **Labour groups: positive** Favourable references to all labour groups, the working class, and unemployed workers in general. Support for trade unions and calls for the good treatment of all employees, including: • More jobs; • Good working conditions; • Fair wages; • Pension provisions etc

The source is the Manifesto Project Database (Volkens et al 2020), drawing on Budge and Laver (1992)

7, and

**Table 8:** First step IV estimates

	(1)	(2)	(3)
*Instruments*			
Quality of institutions (log)	1.157***	1.158***	1.125***
	(0.024)	(0.024)	(0.024)
RILE	−0.083***		
	(0.035)		
Extreme Left		0.079***	0.054***
		(0.020)	(0.021)
Extreme Right			−0.110***
			(0.015)
Digital Tech. (log)	1.211***	1.211***	1.206***
	(0.031)	(0.031)	(0.031)
Population (log)	0.353***	0.351***	0.355***
	(0.009)	(0.009)	(0.009)
Value Added Manuf. (log)	0.004	0.009	0.004
	(0.014)	(0.014)	(0.014)
Dirty Units (log)	−0.002	−0.003	−0.003
	(0.002)	(0.002)	(0.002)
Intermediate	−0.213***	−0.215***	−0.216***
	(0.021)	(0.021)	(0.021)
Rural	−0.323***	−0.327***	−0.330***
	(0.022)	(0.022)	(0.021)
Pre-sample Mean GHG (log)	0.004***	0.004***	0.004***
	(0.001)	(0.001)	(0.001)
Year Dummies	Yes	Yes	Yes
# Observations	10,510	10,510	10,510
Adjusted R^2^	−30,938.38	−30,938.26	−30,935.70
F-test on Instruments	1152.30***	1157.90***	791.40***

Model (2) is the only one that passes the over-identification restriction tests. Robust standard errors in parentheses, clustered at metroregion-level: ***, **, *, indicate significance at the 1%, 5% and 10% level, respectively

8.

## Footnotes

1 The EU’s Industrial Strategy is strongly integrated with other major European initiatives, most notably the *European Green Deal*, aimed at making the EU environmentally sustainable, and the *European Digital Strategy*, aimed at improving the global competitiveness of the EU digital ecosystem. The twin transition is also central to the EU’s long-term budget (the *Multiannual Financial Framework* 2021–2027) and the *NextGenerationEU*, the temporary instrument designed to boost recovery from the coronavirus pandemic.

2 Most of these studies (including the paper at hand) measure technology by means of patent indicators, assuming that patent applications are a good proxy of inventive activities and, consequently, of innovation and its diffusion within a given place or area. While there is no perfect measure for innovation, patent applications, despite obvious limitations, are a reliable proxy for measuring regional innovation (Acs et al. 2002), based on the assumption that they serve as a proxy for innovation inputs entering a given geographical area. At the same time, however, a number of widely accepted taxonomies for identifying patent applications that lead to a reduction in environmental pressures have been developed, most notably the OECD ENVTECH. Indeed, several studies have, on this basis, been better able to identify the role of technology (T) and measure its impact through environmental rather than generic patent applications. We adopt the same approach here. A valuable alternative would have been to measure innovation through a count indicator retrieved from survey data. Such a measure would be more appropriate for capturing the innovation activities of firms that systematically tend not to patent their inventions, although at the cost of it being a subjective and self-reported measure. Yet, the lack of information on fine-grained geographical location of available innovation surveys makes such a data source not applicable to the setting of this study.

3 By way of example, recent estimates suggest that the training pipeline of a single deep learning model for complex natural language processing (NLP) tasks can produce five times the emissions of an average car over its lifetime, which amount to around 300,000 kg of carbon dioxide equivalent (Strubell et al. 2019).

4 The approach we adopt is an extension of the IPAT model employed by Costantini et al. (2017). These authors propose a sectoral analysis of the impact of environmental technology on environmental performance, where the latter is a function of affluence, that is, the level of output scaled by the number of employees (or labour productivity), the state of technology (scaled by the number of employees) and other specific structural fixed effects, including inter-sectoral relatedness.

5 The metropolitan regions correspond to NUTS 3 regions or combinations of NUTS 3 regions constituting agglomerations of at least 250,000 inhabitants. These agglomerations were identified using the Eurostat and European Commission DG REGIO classification based on Urban Audit’s Functional Urban Area (FUA), which are “functional economic units” based on density and commuting patterns of the smallest administrative units for which national commuting data are available (NUTS 3 level in Europe). NUTS 3 regions with more than 50% of the population living within a given agglomeration are aggregated in a metropolitan region. These criteria allow us to relax the limitations associated with the adoption of merely administrative criteria, as would be the case when using the pure NUTS classification which neglects the actual agglomeration of economic activities.

6 Production activities account for about half of all EU GHG emissions. In 2020, for instance, 26% of total EU emissions were attributable to the energy supply macro-sector and 22% to industry (EEA 2021).

7 In general, note the absence of locally measured air emissions from the data. Among other available data sources, we considered the possibility of exploiting three models: namely, EMEP—the Cooperative Program for the Monitoring and Evaluation of the Long Range Transmission of Air Pollutants in Europe; EDGAR—the emission database for global atmospheric research; and COPERNICUS—the European Union’s Earth observation programme. However, the nature of our research requires data on *real* emissions originating from industrial activities that present a certain variability in time and space due to possible technological improvements. In contrast, the aforementioned models are based on assumptions that *project* national data at the local grid level. For instance, in EDGAR, aggregate national emissions are estimated based on the latest available global statistics and, then, reported at the grid level annually depending on country-specific activity, emission factors and technology mix as well as on reduction factors for the abatement systems installed in each sector, with data being drawn from different combined sources, including energy consumption, satellite information, gas extraction, oil consumption and, for certain sectors, from the E-PRTR itself (for detailed information on EDGAR, see Crippa et al. 2018). As it is based on a grid-level projection of national emission data, the use of this dataset here would not satisfy our research needs, as variation (if any) at the local level could not be attributed to changes in the localised knowledge base of a region but rather to changes at the national level. The same reasoning applies to the other alternative models listed above.

8 This trend is broadly consistent with the overall GHG emission data for industry in Europe (EEA 2021).

9 As a robustness check, we restricted our analysis to patent applications in environmental domains related only to the emission of pollutants, by removing for instance waste or water-related applications. Results are consistent with those presented herein.

10 Other technologies, such as 5G networks or blockchain, could have been considered as part of the eco-system. However, our empirical analysis stops in 2016, when these technologies had yet to appear or were in the very early stages of their life cycle.

11 Figures 8 and 9 in Appendix A show the evolution of the specific macro-categories making up the digital ecosystem.

12 Data do not allow to flag energy intensive sectors in a fine-grained way, as the sectoral classification in the data is at two-digit. *Dirty Units* thus counts as energy intensive all units belonging to the manufacturing sectors: Food (C10); Steel and all the basic metals (C24); Chemicals fertilizers and all the manufacture of chemicals (C20); Cement, Lime, Ceramics, Glass and non-metallic mineral products (C23); and paper (C17).

13 As a robustness check, we also included a variable that accounts for the stringency of environmental policies in the countries of interest, using the OECD “Environmental Policy Stringency” composite indicator (country-level). Since the variable showed no significant effect, it was removed for the sake of parsimony. In addition, we tested specifications that include GDP per capita instead of the share of value added in the manufacturing sector (the two being highly correlated). Finally, we also considered models with the dependent variable constructed using the 3-year moving average, as with patent applications. The results were all in line with those discussed here.

14 The results shown and discussed below are insensitive to different specifications, that is the sequential inclusion and/or exclusion of various controls. However, note that the inclusion of country or regional dummies absorbs the effect of *all* coefficients.

## Declarations

### Disclaimer

The scientific output expressed here does not imply a policy position of the European Commission. Neither the European Commission nor any person acting on behalf of the Commission is responsible for the use which might be made of this study.
